# A multicentre simulation study of planar whole-body bone scintigraphy in Sweden

**DOI:** 10.1186/s40658-022-00435-5

**Published:** 2022-02-14

**Authors:** Agnetha Gustafsson, Eva Örndahl, David Minarik, Kerstin Cederholm, Sophia Frantz, Jessica Hagerman, Lena Johansson, Johan Fredén Lindqvist, Cathrine Jonsson

**Affiliations:** 1 Department of Medical Radiation Physics, and Department of Health, Medicine and Caring Sciences, Linköping University, Linköping, Sweden; 2 Equalis AB, Uppsala, Sweden; 3 Radiation Physics. Skåne University Hospital, Lund University, Malmö, Sweden; 4 Department of Radiology, County Hospital Sundsvall-Härnösand, Sundsvall, Sweden; 5 Clinical Physiology and Nuclear Medicine Unit, Department of Translational Medicine, Lund University, Malmö, Sweden; 6 Department of Clinical Physiology and Nuclear Medicine, Skåne University Hospital, Lund, Sweden; 7 Department of Image and Functional Medicin, Central Hospital, Karlstad, Sweden; 8 Department of Clinical Physiology, Sahlgrenska University Hospital, Göteborg, Sweden; 9 Department of Medical Radiation Physics and Nuclear Medicine, Karolinska University Hospital, Stockholm, Sweden

**Keywords:** Multicentre survey, Bone scintigraphy, Image quality assurance, Detection degree, Monte Carlo

## Abstract

**Background:**

Whole-body bone scintigraphy is a clinically useful non-invasive and highly sensitive imaging method enabling detection of metabolic changes at an early stage of disease, often earlier than with conventional radiologic procedures. Bone scintigraphy is one of the most common nuclear medicine methods used worldwide. Therefore, it is important that the examination is implemented and performed in an optimal manner giving the patient added value in the subsequent care process. The aim of this national multicentre survey was to investigate Swedish nuclear medicine departments compliance with European practice guidelines for bone scintigraphy. In addition, the effect of image acquisition parameters on the ability to detect metabolic lesions was investigated.

**Methods:**

Twenty-five hospital sites participated in the study. The SIMIND Monte Carlo (MC) simulation and the XCAT phantom were used to simulate ten fictive patient cases with increased metabolic activity distributed at ten different locations in the skeleton. The intensity of the metabolic activity was set into six different levels. Individual simulations were performed for each site, corresponding to their specific camera system and acquisition parameters. Simulated image data sets were then sent to each site and were visually evaluated in terms of if there was one or several locations with increased metabolic activity relative to normal activity.

**Result:**

There is a high compliance in Sweden with the EANM guidelines regarding image acquisition parameters for whole-body bone scintigraphy. However, up to 40% of the participating sites acquire lower count density in the images than recommended. Despite this, the image quality was adequate to maintain a stable detection level. None of the hospital sites or individual responders deviated according to the statistical analysis. There is a need for at least 2.5 times metabolic activity compared to normal for a lesion to be detected.

**Conclusion:**

The imaging process is well harmonized throughout the country and there is a high compliance with the EANM guidelines. There is a need for at least 2.5 times the normal metabolic activity for a lesion to be detected as abnormal.

## Background

Whole-body bone scintigraphy constitutes a cornerstone of nuclear medicine imaging procedures. Nowadays, Single Photon Emission Computed Tomography combined with Computed Tomography (SPECT/CT) is commonly used as a valuable complement to whole-body bone imaging, especially in doubtful cases. Together, they constitute a highly sensitive diagnostic nuclear medicine procedure where metabolic changes can be detected very early, often several weeks or even months before they become apparent with conventional radiological procedures [1]. In Sweden, bone scintigraphy is one of the most common nuclear medicine examinations, following Fluorodeoxyglucose Positron Emission Tomography (FDG-PET) and myocardial scintigraphy [2]. Therefore, bone scintigraphy is a natural part of the quality assurance (QA) program in nuclear medicine offered nationwide by Equalis AB [3] in Sweden. Equalis AB is a non-profit company providing external quality assessment of laboratory investigations within Swedish health care. Within this program, the goal is to ensure the quality of the entire nuclear medicine process, i.e., from referral, preparation, examination, interpretation and report. Another goal of Equalis QA program is to harmonize nuclear medicine examinations in Sweden. Patients should be able to expect a concordant reading from any nuclear medicine site.

It is challenging to cover all parts of an examination into one single QA program. Conducting written surveys is an easy way to get an overview of used parameters. It allows the sites to see how other departments perform examinations and whether they harmonize with guidelines. The European guidelines on bone scintigraphy [4] is a valuable reference to lean toward. However, surveys do not give any added value when assessing the resulting image quality and the associated report.

Physical phantom studies are useful for testing camera system performance, verifying data acquisition and quantification since the ground truth is known. However, due to the nature of physical phantoms, only generalized geometries can be investigated. The results obtained from such studies may not be directly applicable into a clinical situation. The distribution of a physical phantom in a large multicentre study is also cumbersome and resource-consuming. The pre-measurement preparation that needs to be performed on site could introduce additional uncertainties [5]. Nevertheless, physical phantoms have been used in several multicentre studies by Heikinnen [6–8]. In equivalence to physical phantom studies, results obtained from image data analysis based on MC-simulations can be compared to a known truth. MC simulations aided by anthropomorphic computer phantoms [9] have previously been used in several multicentre studies [5, 10]. The combination of a realistic phantom, with the capability of including respiratory and heart-beating motion patterns, and an accurate scintillation camera simulation package allows for complex, patient-like studies where realistic activity distributions also can be accounted for.

This work presents a national multicentre survey investigating whether Swedish nuclear medicine departments (hereafter called sites) follow the European guidelines regarding imaging parameters for whole-body bone scanning. Furthermore, an investigation was done to investigate whether these parameters affect the assessment of the detection of ten different locations of increased metabolic activity in the skeleton based on simulated image data. This study was performed as a part of a national QA program in nuclear medicine, initiated and managed by Equalis AB.

## Methods

All twenty-eight nuclear medicine sites performing whole-body bone scans in Sweden 2017 were invited to participate in this study. Twenty-five of these accepted the invitation by completing a form providing information on their imaging system, acquisition protocol for whole-body bone scan and the administrated activity routinely used. The acquisition parameters were compared to parameters recommended by the EANM guidelines [4]. SPECT/CT was not included in this study.

### Monte Carlo simulations

Each participating site reported the method used for whole-body scanning, e.g., the camera system, camera settings (energy window, matrix size, pixel size, scan speed, collimator), the administered activity and the time interval between the injection and the scanning. Values for the crystal thickness, energy resolution and the intrinsic spatial resolution were taken from each vendor’s specification. The SIMIND MC program [11] and the XCAT anthropomorphic computer phantom [9] were then used to simulate whole-body bone scan data. Simulations were performed for each site using their specific camera settings. The virtual phantom corresponded to a 180 cm tall male that weighs 85 kg. The bone-to-background ratio of the activity concentration in the phantom was set to 85:1, based on clinical and simulation experience. This yielded a contrast ratio between bone and background in the simulated anterior and posterior images that lies in the range for a patient with normal functioning kidneys and of the same size as the phantom. The bone-to-kidney activity concentration ratio was set to 4:1, also based on clinical and simulation experience. To mimic real measurements the simulations were performed with sufficient histories to generate noiseless data. Poisson noise was added after the simulations, corresponding to the count level representative for the administrated activity and acquisition time used at each site.

**Fig. 1:**
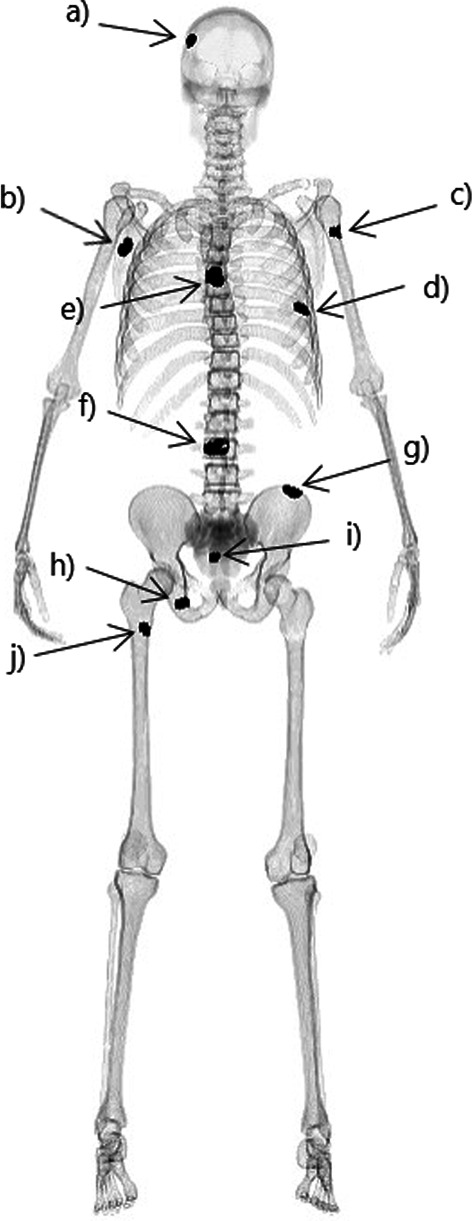
The image shows the original XCAT phantom with skeletal uptake and maximum metabolic activity in the specified locations **a** the cranium, **b** scapula, **c** humerus, **d** the rib, **e** thoracic vertebral column, **f** lumbar vertebral column, **g** os ileum, **h** os pubis, **i** sacrum and **j** femur diaphysis

Ten fictive cases were simulated using the same XCAT virtual phantom for all cases. Each case had ten different locations of increased metabolic activity and each location had a specific volume (Fig. 1), chosen by experienced physicians to cover common locations in clinical bone scintigraphy. The metabolic activity ratio, i.e., the ratio between increased metabolic activity and normal metabolic activity in bone was set to six different levels between 1.0 and 3.5. The locations, the volumes and the distribution of the metabolic activity ratios are given in Table 1.

**Table 1:** The different locations, the volumes and the corresponding ratio between increased metabolic activity normal metabolic activity in bone

Location	Volume (cm^3^)	Case number									
		1	2	3	4	5	6	7	8	9	10
a) The cranium	2.9	1.5	1.0	1.0	3.5	2.0	3.0	1.0	2.5	2.5	1.0
b) Scapula	6.0	1.0	2.0	1.0	3.0	1.0	3.5	1.0	2.5	1.0	1.5
c) Humerus	3.5	1.5	2.0	1.0	3.5	1.0	3.0	1.0	1.0	2.5	1.0
d) Rib	4.6	1.0	1.0	1.0	3.0	1.5	3.5	1.0	2.0	2.5	1.5
e) Thoracic vertebral column	13.0	1.0	2.5	1.5	3.5	2.0	3.0	1.0	3.0	3.0	1.0
f) Lumbar vertebral column	13.5	1.5	2.5	1.0	3.5	1.0	3.0	1.0	2.5	3.5	2.0
g) Os ileum	6.3	1.5	1.0	1.0	3.5	1.0	3.0	1.0	2.5	1.0	2.0
h) Os pubis	5.0	1.5	2.5	1.5	3.5	2.0	3.0	1.0	1.0	3.0	2.5
i) Sacrum	2.6	1.0	2.0	1.0	3.5	1.5	1.0	1.0	2.5	3.0	1.0
j) Femur diaphysis	4.8	1.0	1.0	1.0	3.0	1.0	3.5	1.0	2.0	2.5	1.5

The final whole-body scans were converted into DICOM-format and sent to the respective site to be imported into their evaluation software, in total ten cases were sent to each participating site.

### Image analysis and evaluation

All clinical professions in the nuclear medicine sites were encouraged to participate in the study, here called individual responses. All sites were also encouraged to report a clinical response, i.e., a joint agreement in the clinic.

In the image evaluation study, the participants at each hospital were asked to perform a visual evaluation of their specific simulated cases and report the result in terms of increased metabolic activity relative to normal metabolic activity in the skeleton. No medical history was included. A response protocol was attached to each case. Findings of locations with increased metabolic activity were to be marked with a small circle, at each location where an increased metabolic activity level was found. The participants were informed that there were no limitations in the number of locations with increased metabolic activity. This resulted in ten response protocols, from each participant. The reported findings were compared to the true values in each location. The number of protocols containing false positive findings were compared to the total number of protocols. Each participant also had to answer a questionnaire about profession, degree of education, number of years in the profession and if they sign clinical assessments of bone scan examinations independently.

### Statistical analysis

Statistical analysis of the results was carried out using logistic regression [12] where the correct answer was used as the dependent variable and including all categorical variables as the answer from the responders, i.e., the sites, type of responses, cases, metabolic activity locations and volumes, professions, gamma camera type and continuous variables as pixel size and count density. A *p* value < 0.05 was considered to be statistical significant. The result from the logistic regression was also used to calculate a receiver operating curve (ROC) (12) using IBM^@^ SPSS^@^ Statistics version 25. The sensitivity and specificity were calculated based on the answer from the responders compared to the correct answer.

## Results

The number of nuclear medicine sites that answered the acquisition parameter survey were twenty-five. A few sites reported parameters from more than one gamma camera system. The reported acquisition parameters from the different sites, used in the Monte Carlo simulations, are listed in Table 2, together with the recommendations from EANM. Overall, the compliance with the EANM guidelines is good. Deviations were found in one site that uses a broader energy window than recommended and three sites differed from the recommendation on scan velocity. However, there was a remarkable deviation from the EANM guideline for the total number of counts in the final images. The anterior counts from all simulated images ranged from 0.9 to 2.4 Mcounts, and the posterior counts ranged from 1.0 to 2.9 Mcounts. Only 15 of 25 sites fulfilled the recommendation of at least 1.5 Mcounts in both the anterior and posterior image. Case number 7 resulted in the lowest number of counts for both anterior and posterior images. As expected, there was little difference in total number of counts between the different cases for a specific gamma camera system.

**Table 2:** The reported image parameters used in the Monte Carlo simulation and the number of total counts in the anterior and the posterior bone scan image from each hospital site

Site number	Energy window (%)	Centre of energy window (keV)	Injected activity (MBq)	Matrix-X	Matrix-Y	Scan velocity (cm/min)	Pixel size (cm)	Gamma camera system	Collimator	Time between injection and imaging (h)	Total number of counts	
											Anterior	Posterior
	(15–20%)*	(140–143)*	(300–740)*	(256–512)*	(1024)*	(10–15)*	(no,. recommendation)*		(LEHR)*	(2–5)*	(> 1,500,000)*	(> 1,500,000)*
1	21%	141.0	490	256	1024	15	0.22	GE 670	LEHR	2,5	1,100,816	1,313,060
3	21%	140.5	550	512	1024	10	0.20	Philips Brightview XCT	LEHR	3	1,931,430	2,303,850
7	21%	145.5	570	512	1024	15	0.20	Philips Precedence	LEHR	3	1,338,693	1,595,504
13	16%	143.0	500	256	1024	13	0.23	Siemens Symbia T2	LEHR	2	1,389,344	1,656,818
21	21%	140.5	600	512	1024	12,5	0.20	Philips Brightview XCT	LEHR	3	1,693,839	2,018,439
26	11%	141.0	550	256	1024	12	0.24	GE 670	LEHR	3	1,073,895	1,287,131
31	31%	140.5	500	256	1024	12	0.22	Siemens Symbia T16	UHR	3	877,851	1,043,091
33	16%	143.0	590	256	1024	12	0.24	Siemens Symbia True Point	LEHR	3	1,588,112	1,893,950
38	18%	141.0	600	256	1024	10	0.22	GE 640	LEHR	4	1,685,911	2,007,754
38	18%	141.0	600	256	1024	10	0.22	GE 670	LEHR	4	1,692,886	2,016,032
38	18%	141.0	600	256	1024	10	0.22	GE 670	LEHR	4	1,693,896	2,016,601
43	16%	143.0	550	256	1024	9	0.24	GE 640	LEHR	3	1,734,221	2,075,107
46	21%	140.5	550	256	1024	10	0.22	GE 670	LEHR	3.5	1,649,906	1,966,067
46	21%	140.5	550	256	1024	10	0.22	GE Infinia	LEHR	3.5	1,993,627	2,372,651
61	18%	142.3	500	256	1024	10	0.22	GE Infinia	LEHR	3	1,756,555	2,100,451
68	15%	140.5	500	256	1024	17	0.24	Siemens Intevo	LEHR	2.5	981,228	1,170,397
87	21%	140.5	570	256	1024	12	0.33	Philips Brightview XCT	LEHR	3	1,673,813	1,992,612
90	21%	140.5	500	256	1024	12	0.21	GE Infinia	LEHR	2.5	1,688,770	2,013,362
95	21%	140.5	500	256	1024	10	0.22	Infi nia	LEHR	3	1,919,824	2,286,357
97	31%	140.5	550	256	1024	15	0.24	Siemens	LEHR	4	1,347,915	1,599,962
99	18%	141.0	550	512	1024	12	0.28	Phyilips SkyLight	LEHR	3.5	1,369,623	1,630,865
99	18%	141.0	550	512	1024	12	0.28	Phyilips SkyLight	LEHR	3.5	1,367,211	1,631,325
110	16%	143.0	600	256	1024	15	0.24	Siemens Symbia	LEHR	4	1,150,123	1,369,556
112	18%	141.0	550	512	1024	12	0.28	Philips Brightview	LEHR	3	1,600,004	1,904,257
118	18%	142.3	550	256	1024	8	0.22	GE Infinia	LEHR	3	2,419,319	2,890,270
123	16%	140.0	550	256	1024	12	0.24	Siemens Symbia T16	LEHR	3	1,498,083	1,786,826
129	14%	141.0	550	256	1024	10	0.19	Siemens Symbia Intevo 2	LEHR	3	1,685,846	2,016,157
135	16%	143.0	600	256	1024	12	0.22	GE 670	LEHR	2.5	1,503,300	1,796,332
73	16%	143.0	500	256	1024	15	0.22	GE NM/CT 640	LEHR	3	946,471	1,127,901
73	16%	143.0	500	256	1024	15	0.24	Siemens Symbia T6	LEHR	3	1,059,915	1,268,153

The site numbers are randomized

*The EANM recommended values for the different parameters are given in brackets

Twenty-three sites answered the image evaluation study. The number of individual responses were in total 65, and the distribution between professions and their experience is shown in Table 3. Eleven clinical responses were submitted. The result from the statistical evaluation of the image evaluation study using logistic regression shows that no statistical differences could be indicated with a 95% confidence, in the results between different sites. Among the individual responses, there was not either any statistical differences. There were no statistical differences between clinical responses and individual responses, nor between the different professions. Furthermore, no statistical differences were obtained when considering the count density in the images or the different volumes of increased metabolic activity. Nor was there any statistical difference, using different acquisition parameters, such as type of gamma camera or pixel sizes in the images.

**Table 3:** The number of responses and the distribution between different professions

Total number of responses	76
Experienced medical doctors	32
Not experienced medical doctors	6
Nurse/technologists	24
Physicists	3
Clinical responses	11

The statistical analysis using ROC for the method whole-body scintigraphy, resulted in an area under the curve (AUC) 0.885 with a confidence interval of 0.878–0.892. The calculated values for the sensitivity, the specificity and AUC based on logistic regression is shown in Table 4. The result shows that the AUC-value increases as the low activity levels are excluded in the calculation, confirming the increase in sensitivity when considering higher uptake lesions.

**Table 4:** The values for the sensitivity, specificity and the AUC, for the whole-body scintigraphy method including different activity levels in the calculation

Number of locations	Activity levels included	Sensitivity (%)	Specificity (%)	AUC
7597	1, 1.5, 2.0, 2.5, 3.0, 3.5	51	94	0.885
6609	1, –, 2.0, 2.5, 3.0, 3.5	80	94	0.953
5849	1, –, –, 2.5, 3.0, 3.5	73	94	0.988
4862	1, –, –, –, 3.0, 3.5	81	94	1.000
3875	1, –, –, –, –, 3.5	82	94	1.000

**Fig. 2:**
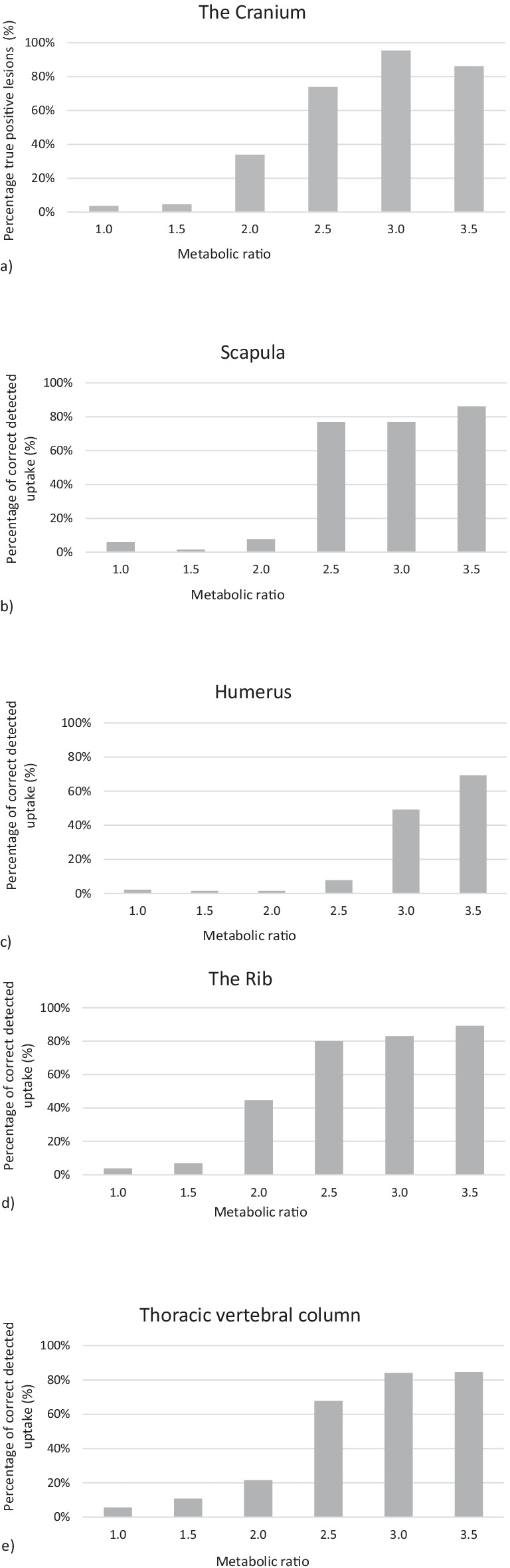

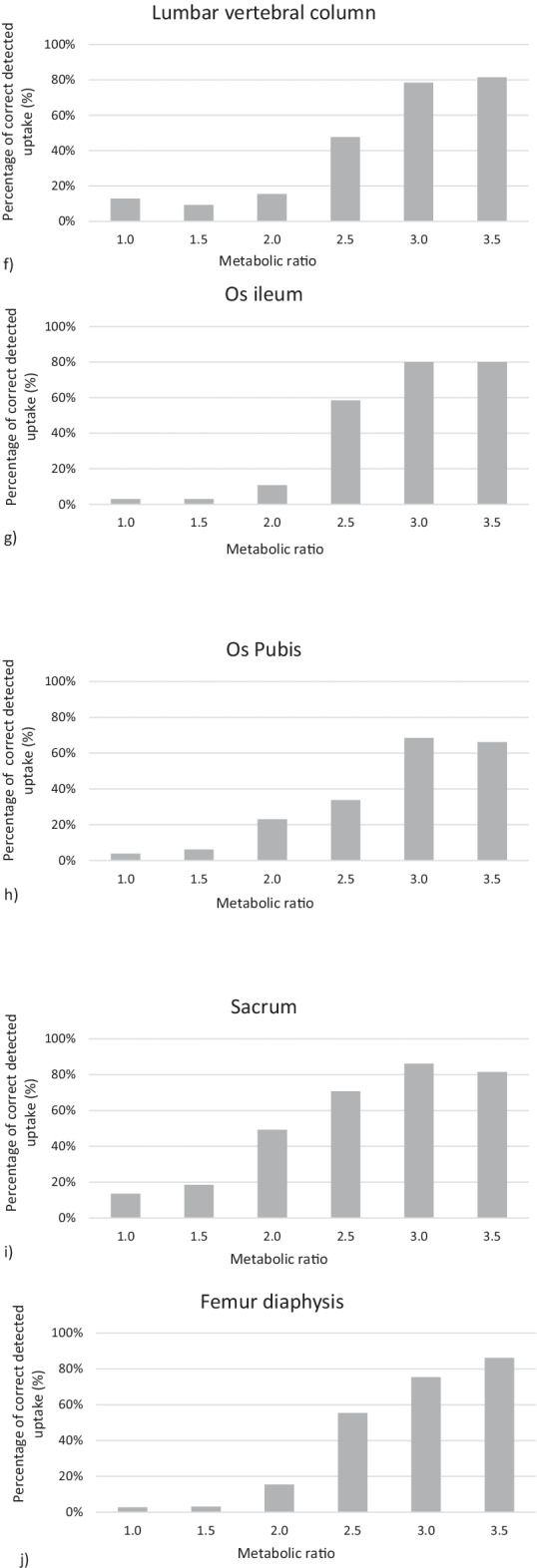
The percentage true positive lesions plotted against the ratio between the increased metabolic activity level compared to normal activity in the skeleton, for the ten different locations **a** the cranium, **b** scapula, **c** humerus, **d** the rib, **e** thoracic vertebral column, **f** lumbar vertebral column, **g** os ileum, **h** os pubis, **i** sacrum and **j** femur diaphysis

The result of the image evaluation study for all the individual responses is displayed in Fig. 2, for each location. There is a clear limit at intensity 2.5 times the normal activity where a majority of the participants reported the increased metabolic activity. More than 60% of the participants reported the increased activity localized in the cranium, scapula, the rib, the thoracic vertebrae column, and sacrum. In the lumbar vertebra column and in the os pubis, 3.0 times as high metabolic activity than in normal skeleton is required in order to be detected by more than 60% of the participants. In the humerus, the corresponding increase in metabolic activity required was 3.5.

**Fig. 3:**
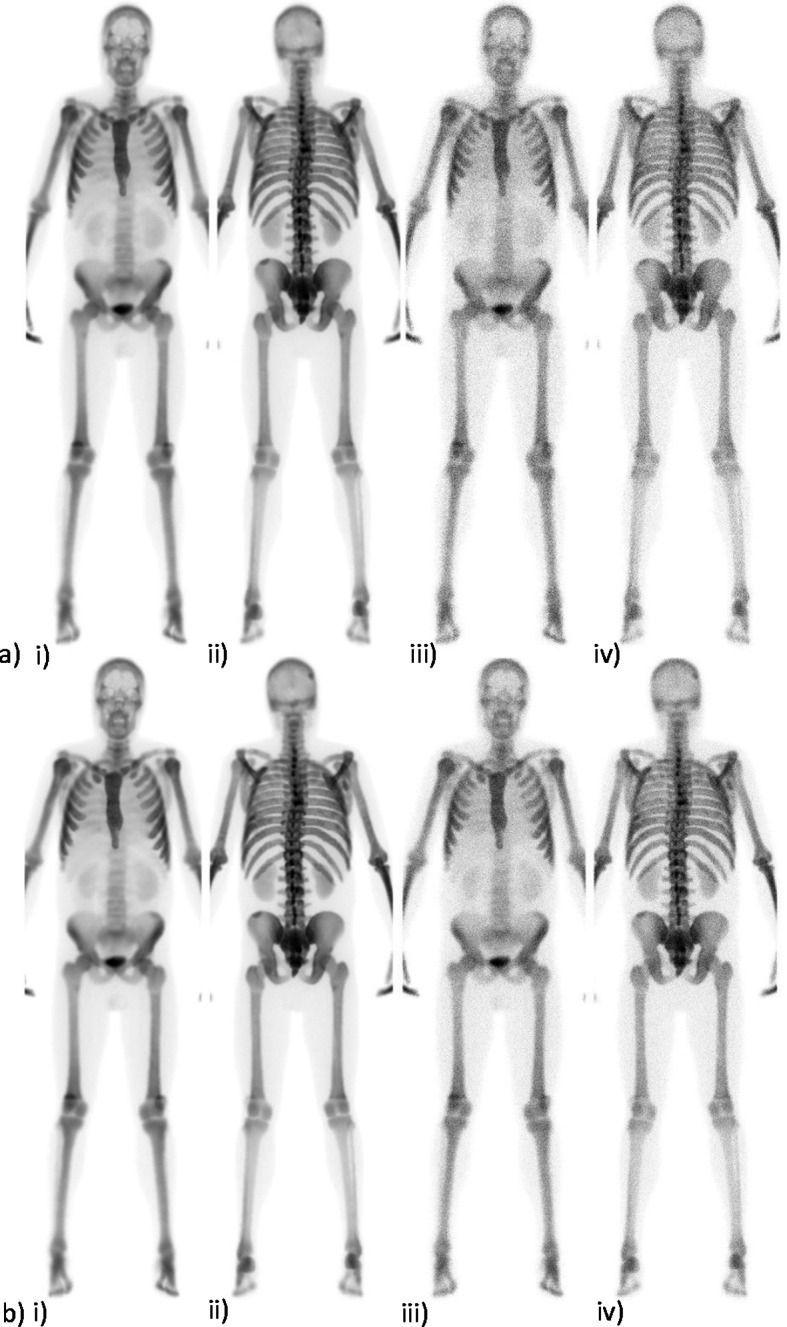
Four simulated whole-body scans for patient case eight are shown in **a** images from site number 1 and in **b** images from site number 118. In image i) the anterior view and image ii) the posterior view without noise shown. In image iii) the anterior view and in image iv) the posterior view with noise is shown

In Fig. 3, the simulated patient case number eight is shown for sites 1 and 118, both with and without added Poisson noise. This patient case has increased metabolic activity in all locations except in the humerus and os pubis. Site number 118 detected all locations with increased metabolic activity and site number 1, did not detect any of these, in their respective clinical responses. Among the individual responses, there were 32–55% of the reports, that contained one or several false positive findings. Among the clinical responses, the corresponding range was 9–27%. The proportion of false positive findings in the clinical responses assessed on images containing 1.5 Mcounts or more were 26% compared to 15% in the responses assessed on images containing counts less than 1.5 Mcounts.

## Discussion

Our study shows that the compliance with the EANM guidelines is high in a majority of the nuclear medicine sites in Sweden. No clinic had deviating results regarding the image evaluation study. The imaging process is harmonized throughout the country and there is no doubt that bone scintigraphy is a valuable method for detection of increased metabolic activity in the skeleton. However, the results from the ROC-analysis still show room for improvement.

The image quality is fundamental for clinical assessment of images. Physical characteristics important for the image quality in nuclear medicine are the spatial resolution, the contrast and noise. All these factors depend on the pixel size, the collimator used and the count density in the image determined by the administered activity and the scan speed. In this study, the detectability refers to the ability to detect locations with increased metabolic activity. It is a definition closer to the clinic approach to image analysis and interpretation comparing with the Rose criterion [13], according to which the contrast-to-noise ratio must exceed 3–5 for an object to be detectable.

To assure a “good enough” image quality in whole-body bone scintigraphy, the EANM guidelines are valuable to lean toward. The sites in Sweden follow the guidelines regarding pixel size and the collimator used. However, there was a great deviation from the guidelines regarding the count density in the image, where 40% of the sites acquire a lower number of counts. In the guideline from EANM, 2016 [4], the recommendation is that the scanning speed should be adjusted so that routine anterior and posterior whole-body images each contain more than 1.5 Mcounts. In this study, the total count density, i.e., the sum of the anterior and the posterior images, varied from 1.9 Mcounts to 5.3 Mcounts. For this image evaluation study, the count density did not affect the detection degree, probably due to the way of defining detectability. The ratio between increased metabolic activity and normal metabolic activity in bone was set at fixed levels, Table 1, regardless of the count density. The responders detected locations with increased activity just as well in images with lower count densities. The volumes of the increased metabolic activity differed between locations, as shown in Table 1. It is expected that detectability for the smallest volumes (2.6 cm^3^) will be affected by limited spatial resolution. In this study, the lesion volumes did not significantly affect the detection degree. A study should be designed to vary the different parameters affecting detectability systematically. Consequently, the results from our study therefore do not question EANMS’ recommendations regarding adequate count density, something that is supported by previous studies [14]. Gustafsson et al. performed a visual grading study that resulted in a significant improvement in perceived image quality using an activity level of 600 MBq compared to lower activity levels in whole-body bone scintigraphy.

Following intravenous injection 50% of the 99mTc-diphosphonates accumulate in the skeleton [15]. The factors determining bone uptake of 99mTc-diphosphonate complexes are increased blood flow to the skeleton and reactive bone formation [16, 17]. In the present study, based on MC simulations, the effects from physiological differences in the accumulation mechanism or the time difference between injection and image acquisition are not included. A possible method to improve the detectability of increased metabolic activity is to increase the bone-to-background ratio by lowering the background level in the images. A longer waiting time between injection and image acquisition tends to increase the bone-to-background ratio [4]. All sites in Sweden follow the recommendation from EANM [3] of 2–5 h between injection and image acquisition. By hydrating the patient during the waiting time between the injection and the examination, further optimization is possible according to Starck et al. [18]. The result of their study showed a reduction of number of counts in the background as well as a reduced effective dose to the patients. This reinforces the idea that the detection degree may not strongly be affected by the total image count density but is dependent on the bone-to-background ratio. Thus, the count density in the lesion of interest will influence the detection degree.

It is essential to notice that even these ten cases are fictive, the increased activity was positioned in relevant clinical locations. This study shows that the detection degree of increased metabolic activity in the skeleton using whole-body bone scintigraphy is about 2.5 times the normal activity in bone. The detection degree depends on the location of the uptake, Fig. 2. It seems easier to detect uptake in the rib than uptake in the humerus or os pubis. It is reasonable to make the conclusion that uptake at superficial locations with less surrounding soft tissue, for instance in the rib or the cranium, are less affected by attenuation, will have better contrast, and are easier to detect than corresponding uptakes in deeper locations where there is more surrounding soft tissue, for instance the lumbar vertebrae. This study did not include SPECT/CT, that would possibly have increased the detection degree at deeper locations [19].

The sensitivity in this study turns out to be in the range 51–82% depending on which activity ratios of the lesions were included in the calculation, Table 4. This result is slightly lower compared to the results from a study by Liu et al. [20] where the sensitivity for bone scintigraphy was in the range 74–84%. The lower values in this study are obtained when we include the lowest metabolic ratios, which may not even be clinically relevant, as shown in Fig. 2.

The statistical analysis showed no significant difference between individual and clinical responses regarding false positive findings. The specificity calculated in this study is comparable to results from Liu et al. [20]. However, the number of false positive findings was lower for the clinical responses. We expect that when experienced responders work together with less experienced, valuable learning takes place that could lead to a more correct interpretation. There was no significant difference between the professions regarding either detection of false positives or true positives in this study. However, no medical history was given, i.e., there were no background information available that usually are the case in daily clinical interpretation of whole-body bone scintigraphies, this means that the competence of the physician was not fully taken into account. Moreover, the responders may be biased by assessing previous cases with metabolic activity uptake in corresponding locations. The fraction of false positive findings would probably have been lower if SPECT/CT were included in the study [19].

A limitation with this study was that we only changed the intensity of the lesion at the different locations and not the sizes. As mentioned, the detectability of a lesion should depend in theory on the size, intensity and location of the lesion. By systematically varying these factors, it is possible to evaluate the detectability in a more controlled manner. Since the primary focus with this study was to carry out a multicentre evaluation of image quality, the lesion sizes as well as intensity and location were selected so that the image of the phantom mimicked, to as large an extent as possible, that of an ordinary bone scans, that can be seen in the clinic on a regular basis. Furthermore, this study was based on simulations that is not equal to a real measurement since effects of, e.g., non-perfect intrinsic spatial uniformity and linearity was not modeled. Patient movement was not modeled either. The outcome of this study may therefore be better than if actual patients were included in the study.

## Conclusion

In this study, a digital phantom and MC-simulation method were used to generate ten whole-body bone scintigraphy cases for a multicentre survey in Sweden. The imaging process is well harmonized throughout the country and there is no doubt that bone scintigraphy is a valuable method for detecting increased metabolic activity in the skeleton. There is high compliance with the EANM guidelines regarding imaging parameters. However, up to 40% of the participating sites have lower count density in the images than recommended. There is a need for at least 2.5 times the normal metabolic activity for a lesion to be detected as abnormal.

## Data Availability

The datasets used and/or analyzed during the current study are available from the corresponding author on reasonable request.

## Abbreviations

EANM: European Association in Nuclear Medicine
MC: Monte Carlo
FDG: Fluorodeoxyglucose
PET: Positron Emission Tomography
QA: Quality assurance
SPECT: Single-Photon Emission Computed Tomography
ROC: Receive operation characteristics
DICOM: Digital Imaging and Communications in Medicine
ROC: Receiver operating characteristic
AUC: Area under the curve
